# Supplementary figures and images for: Amoeba predation of Cryptococcus: A quantitative and population genomic evaluation of the accidental pathogen hypothesis
Source: PLoS Pathog. 2023 Nov 13;19(11):e1011763. doi: 10.1371/journal.ppat.1011763 (PMC10681322; doi:10.1371/journal.ppat.1011763)

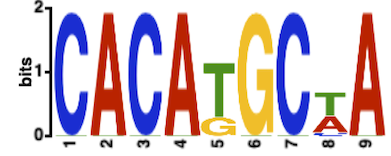

Supplement: S1 Fig — The motif analysis tool XSTREME [55] was used to identify sequence motifs over-represented in 1 kb upstream regions of genes that exhibit expression similar to BZP4. This sequence logo represents a motif found in 9 of 36 upstream regions (E-value 1.56e-008). (TIFF) [file ppat.1011763.s001.tiff]

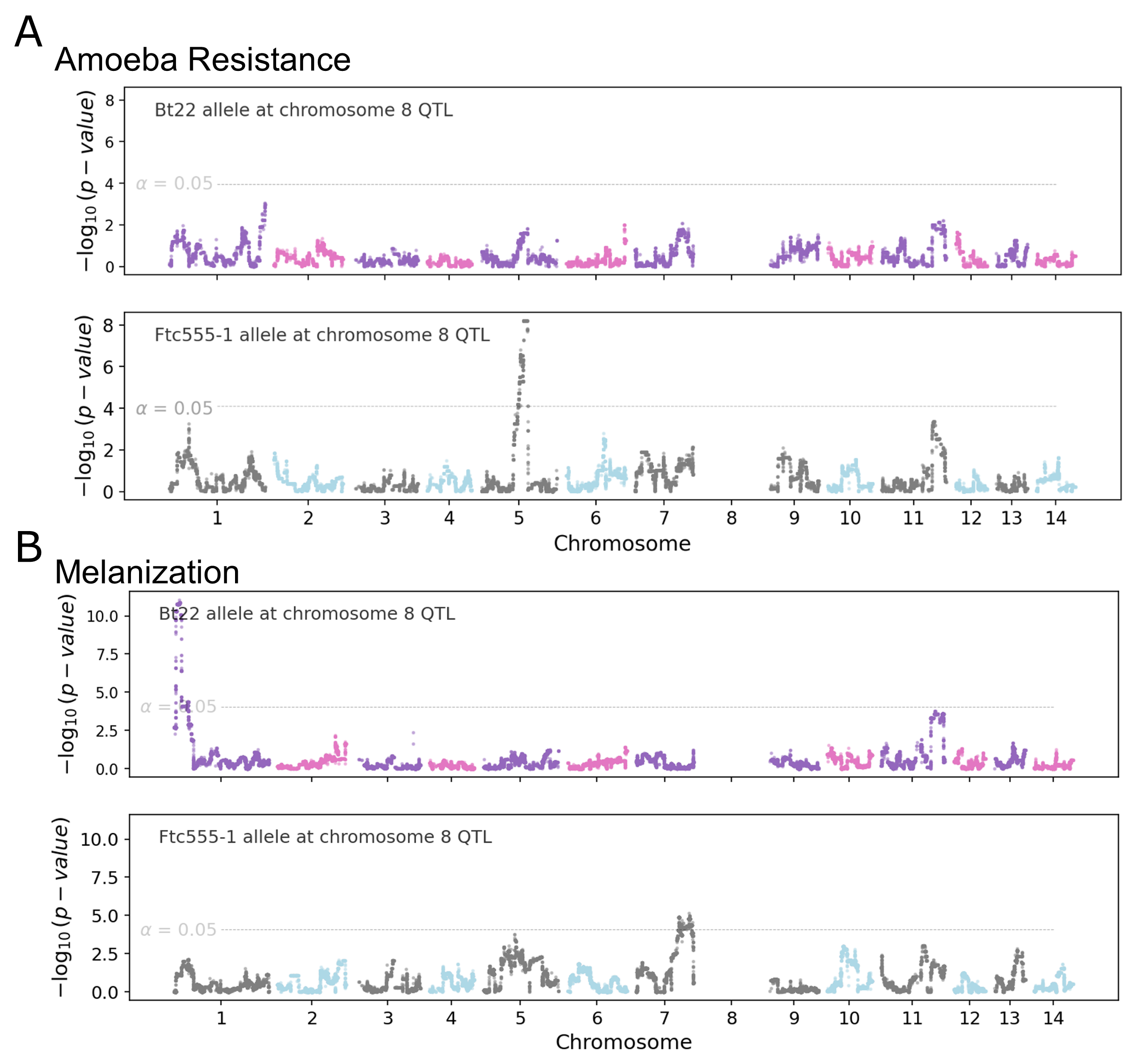

Supplement: S2 Fig — The offspring of the Bt22 × Ftc555-1 cross were subset by genotype at the chromosome 8 QTL, and QTL mapping was repeated for each subpopulation. Variation on chromosome 8 was excluded from consideration. The dotted lines indicate significance thresholds (α = 0.05) determined by permutation testing. A. Manhattan plots for QTL mapping of amoeba resistance, conditional on chromosome 8 QTL genotype. B. Manhattan plots for QTL mapping of melanization, conditional on chromosome 8 QTL genotype. (TIFF) [file ppat.1011763.s002.tiff]

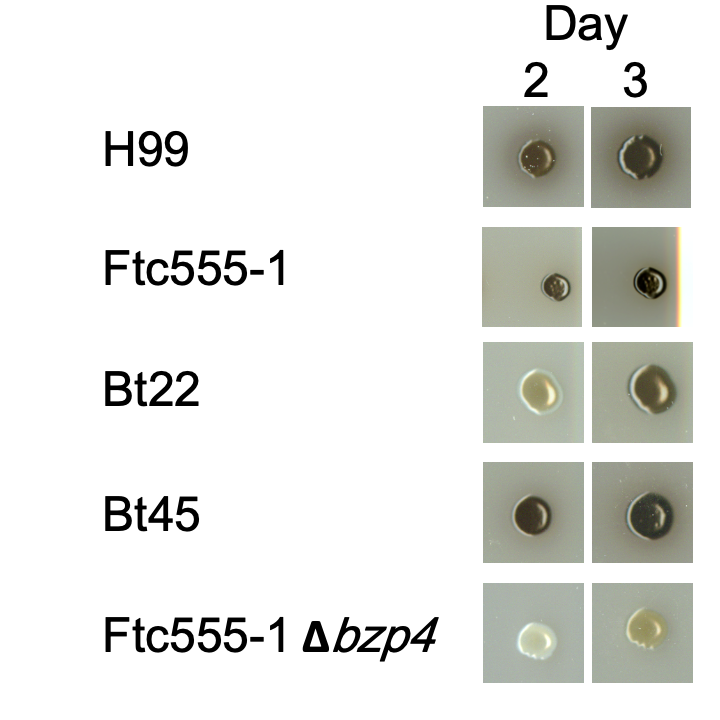

Supplement: S3 Fig — Images of colonies pinned onto L-DOPA plates and imaged after two and three days of growth. The bzp4 deletion mutant and Bt22, which has a 2 kb deletion upstream of BZP4, are slow to melanize but still capable of some degree of melanin synthesis. Images of colonies are uniformly brightened by 30% to better visually contrast the level of melanization. (TIFF) [file ppat.1011763.s003.tiff]

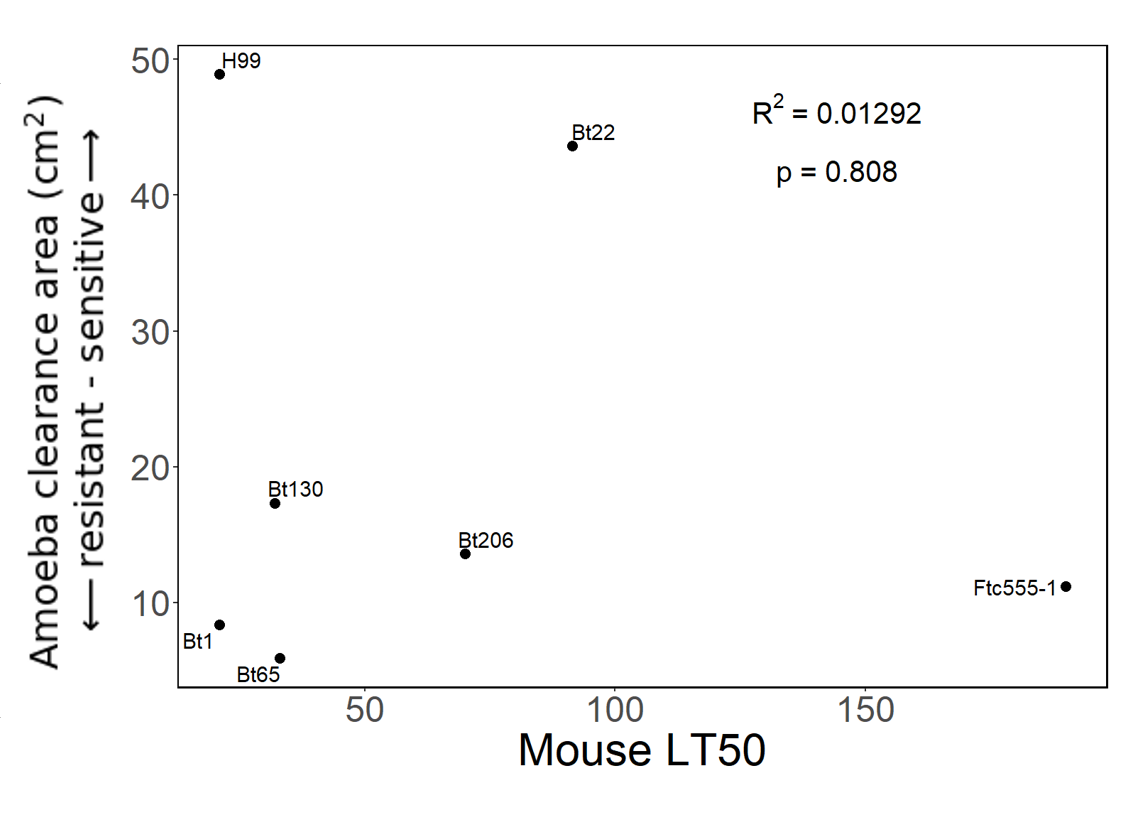

Supplement: S4 Fig — The relationship between amoeba resistance and median time to death (LT50) of mice infected with C. neoformans strains. Significance determined by linear regression. Segregants that were avirulent were assigned a value of 190 days for LT50. (TIFF) [file ppat.1011763.s004.tiff]

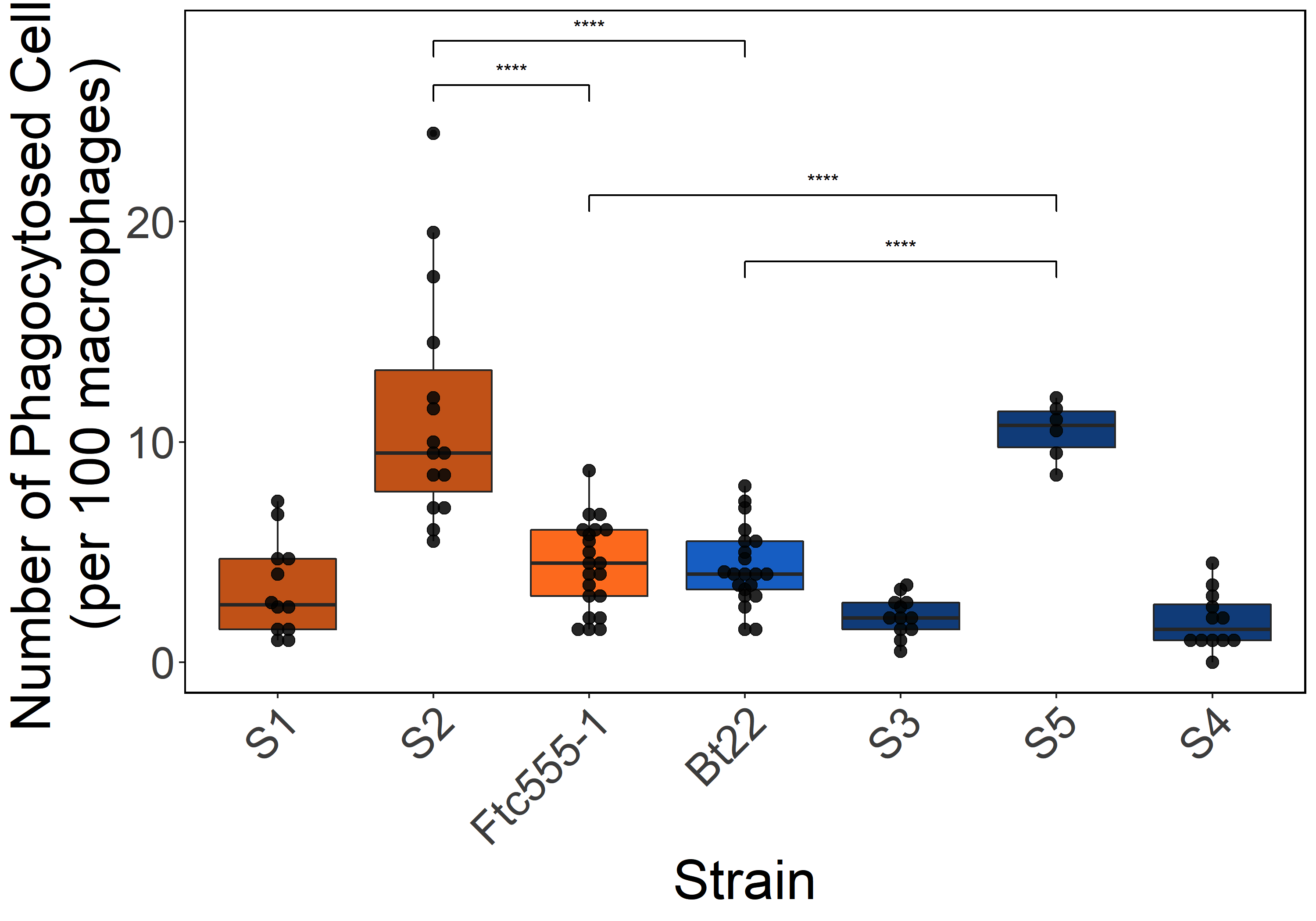

Supplement: S5 Fig — Boxplots representing the phagocytic index of parental strains and segregants. Strains are oriented in rank order of amoeba sensitivity. Boxplots are colored by chromosome 8 QTL genotype. Orange indicates strains with the Ftc555-1 allele and blue those with the Bt22 allele. Significance determined by ANOVA (F = 22.18; p<0.0001). (TIFF) [file ppat.1011763.s005.tiff]
