# Supplementary material for: Amoeba predation of Cryptococcus: A quantitative and population genomic evaluation of the accidental pathogen hypothesis
Source: PLoS Pathog. 2023 Nov 13;19(11):e1011763. doi: 10.1371/journal.ppat.1011763 (PMC10681322; doi:10.1371/journal.ppat.1011763)
Supplement: S4 Table — π and Tajima’s D for BZP4, for each of the three major lineages of C. neoformans compared to other genes on Chromosome 8 and other transcription factors (“control gene sets”). The fifth percentile of the distribution of Tajima’s D in the control gene sets is provided for comparison. The list of predicted transcription factors is from Jung et al. 2015 [51]. (PDF) [file ppat.1011763.s009.pdf]

Table 1: ***BZP4* sequence diversity relative to other transcription factors.** Measures of nucleotide sequence diversity of the *BZP4* coding region compared to the distribution of the same measures for 177 other transcription factors and all other predicted genes on chromosome 8.

Lineage	BZP4 π	Chr 8 Median π	TF Median π	BZP4 Tajima's D	Chr 8 5th percentile Tajima's D	TF 5th percentile Tajima's D
VNI	0.00022	0.0012	0.0012	-1.97	-1.81	-2.06
VNBI	0.00123	0.0027	0.0027	-0.97	-2.03	-2.19
VNBII	0.00106	0.0026	0.0026	-1.14	-2.07	-2.01
